# Chimeric Approach to Identify Molecular Determinants of Nicotinic Acetylcholine Receptors

**DOI:** 10.3390/ijms27021091

**Published:** 2026-01-22

**Authors:** Pooja Sapkota, Seyedeh Melika Akaberi, Biwash Ghimire, Kavita Sharma

**Affiliations:** Department of Biomedical and Pharmaceutical Sciences, College of Pharmacy, Idaho State University, Pocatello, ID 83209, USA; poojasapkota@isu.edu (P.S.); seyedehmelikaakab@isu.edu (S.M.A.); biwashghimire@isu.edu (B.G.)

**Keywords:** nicotinic receptor, acetylcholine, ligand-gated ion channels, chimera, acetylcholine binding protein, recombinant, protein engineering, expression, AlphaFold, computational modeling

## Abstract

Nicotinic acetylcholine receptors (nAChRs) are membrane-bound proteins that mediate fast synaptic transmission throughout the nervous system. A functional nAChR subtype is formed by the combination of multiple subunits arranged as homomeric or heteromeric pentamers, each with a distinct pharmacological profile. Disruption of their neurotransmission contributes to various neuropathologies, emphasizing the need for detailed knowledge of receptor structure, function, subunit composition, dynamics, and potential ligand-binding sites. However, their structural complexity as integral membrane proteins has hindered expression in mammalian cell lines and proven even more challenging to crystallize, limiting insights into ligand interactions. Understanding the molecular determinants governing nAChRs function is essential for the rational design of selective therapeutics targeting neurological disorders. The emergence of a chimeric receptor approach has dramatically improved the ability to study these important proteins and opened new avenues for high-throughput screening in drug discovery efforts. This review explains how the design of chimera constructs using soluble homologs, such as AChBP, provides researchers with an immense opportunity to investigate receptor structure–function relationships and subtype-specific properties, thereby facilitating the development of more effective treatments.

## 1. Introduction

Nicotinic acetylcholine receptors (nAChRs) have been a central focus in the field of neurobiology for several decades, and yet structural determinants underlying their function remain incompletely understood across receptor subtypes [1]. nAChRs are neurotransmitter-gated ion channels that mediate critical physiological functions in both central and peripheral nervous systems, regulating processes such as cognition, motor control, and autonomic functions [2]. Abnormalities in these receptors, resulting from neuronal degeneration, overexpression, or mutations, can lead to diseases for which effective cures are still lacking. Therapeutic management of such pathologies requires structural characterization with high resolution, elucidation of protein–ligand interaction, and knowledge of conformational dynamics [3,4]. Despite their biomedical significance, obtaining high-resolution structural data has been particularly challenging due to their complex membrane-associated structure. This limitation has hindered progress in elucidating their molecular mechanisms and in developing subtype-selective therapeutics [5,6].

Identifying the molecular determinants of nAChRs is challenging due to several factors. These include their nature as transmembrane proteins that require a native lipid environment to maintain their structural integrity and functional stability. Their complex membrane structure is highly flexible and can exist in multiple conformations, including resting, active, or desensitized states [7,8,9]. In addition, nAChRs are typically expressed at low levels on the cell surface and are denatured by many detergents used for solubilizing them [7,10,11]. Their complex heterogeneous pentameric assembly further complicates structural studies, as they require precise stoichiometry and subunit-dependent folding, often necessitating specific molecular chaperones to produce functional surface receptors [12,13]. Together, these factors make it extremely challenging to obtain stable receptors with preserved integrity and purity suitable for high-resolution structural analysis using traditional methods such as X-ray crystallography, nuclear magnetic resonance (NMR), or cryogenic electron microscopy (cryo-EM) [14,15]. These structural biophysical techniques can deduce the internal structure of the protein at atomic resolution; however, expression of highly pure proteins in sufficient quantities, optimizing the correct crystallization conditions, and, especially, the challenges of crystallizing the transmembrane proteins are significant hurdles in structural biology [16,17,18].

To address these persistent structural limitations, scientists have pioneered chimeric receptor constructs as a promising solution. The chimeric receptor strategy is an emerging technique that has facilitated breakthroughs in understanding the biology of nAChRs by revealing their structure and functions, including the agonist binding mechanism, structural rearrangements during signal transduction, and allosteric modulation sites critical for drug design. These are engineered proteins designed by combining parts of different receptors, such as fusing ligand-binding domains of nAChR subtypes with stable and crystallizable structural scaffolds, for structural investigation of binding sites in receptor subunits [19,20,21]. This strategy is a powerful tool for studying the native structure of receptors that are difficult to express or crystallize, as it enhances receptor solubility and stability [22,23].

The utilization of this technique has increased substantially since the early 2000s and has evolved from initial structural characterization to high-throughput fragment screening and allosteric site mapping for ion channel receptors [24,25,26]. The success of this approach depends on preserving the primary functional component, particularly the ligand-binding architecture of the receptor of interest, while conferring the favorable biochemical properties of soluble homologs [23,27]. When co-crystallized with ligands, the chimeric receptor yields stable complexes that provide atomic-level insights into how receptors interact with ligands, revealing structural determinants and molecular rearrangements responsible for ligand recognition, binding affinities, and signal transduction [28,29].

This review describes how difficult-to-express proteins can be engineered by preserving the primary functional components of the receptor of interest while incorporating soluble homologous scaffolds to study the structure–function relationship. It highlights an alternative approach to expressing native nAChRs, which can then be complexed with agonists, antagonists, or allosteric modulators for further structural analysis using crystallography. This approach is crucial for unraveling the structural determinants and molecular rearrangements responsible for ligand recognition and signal transduction in nAChRs.

## 2. Nicotinic Acetylcholine Receptors (nAChRs)

nAChRs have attracted significant attention as a potential drug target because of their extensive presence across the central and peripheral nervous system. They mediate a spectrum of physiological functions ranging from high-order cognitive functions in the brain to critical synaptic transmission at the neuromuscular junction [30]. However, dysregulation of their functions can lead to diverse forms of neurological disorders, including neurodegenerative disorders like Alzheimer’s disease, Parkinson’s disease, neurodevelopmental disorders like autism, psychiatric disorders like schizophrenia, depression, and various autonomic dysfunctions [31,32,33,34,35]. Given these serious implications, understanding the functional architecture of nAChRs has long been the focus of neuropharmacology and structural biology.

nAChRs are ligand-gated ion channels (LGICs) with a pentameric conformation made of multiple subunits. They contain a characteristic loop formed by thirteen highly conserved amino acids between two cysteine residues connected by a disulfide bond in the N-terminal extracellular ligand-binding region. This structural property classifies them as members of the Cys-loop receptor superfamily [36,37]. Acetylcholine is the principal neurotransmitter and a full agonist of nAChRs, while the receptor is also activated by exogenous agonists such as nicotine, cytisine, epibatidine, and carbachol [38]. Agonist binding induces a conformational transition from the closed to the open state, leading to the cation influx through the pore [39]. In the nAChR family, the aqueous ion channel pore is located in the transmembrane domain, and its gating mechanism is guided by the presence of agonists in the ligand-binding site of the extracellular domain [39,40,41]. These receptors are categorized into muscle and neuronal types. The muscle-type nAChRs are heteropentamers located postsynaptically in the neuromuscular junction and assembled from two α1 and one β1, δ, and γ (fetal) or ε (adult) subunits. The neuronal types exhibit a wide distribution across neuronal populations and are even found in some non-neuronal tissues. They can assemble as homomeric types composed solely of α subunits, like α7 or α9, or heteromeric, consisting of both α and β subunits, like α4β2 [42,43,44]. The heteromeric α9α10 nAChRs show restricted expression in the cochlear outer hair cells and have been classified as hair cell receptors with characteristics deviating from the conventional nicotinic types [45,46].

**Figure 1:**
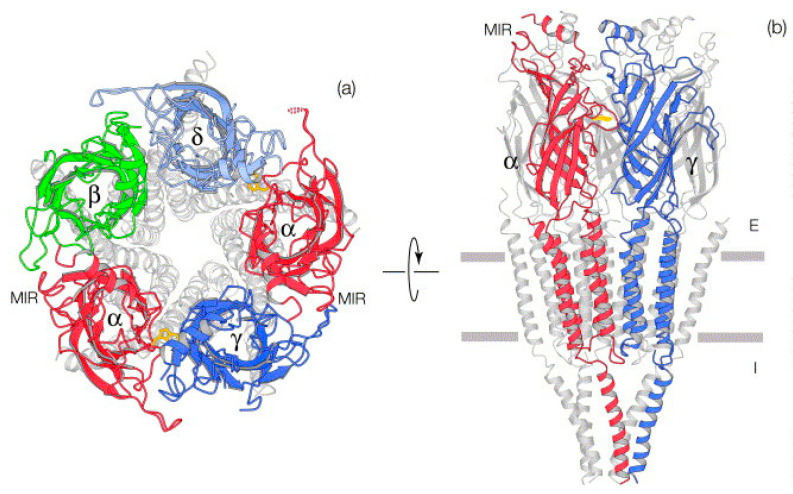
Pentameric structure of *Torpedo marmorata* Acetylcholine Receptor. (**a**) Synaptic cleft view, ligand binding domain is highlighted with distinct colors for each subunit, 2α (red), β (green), δ (light blue), and γ (blue). (**b**) Parallel orientation; front two subunits (α and γ) are highlighted. Extracellular domain (E) consists of N-terminal α-helix and β-sheets (αTrp149 shown in gold), Transmembrane Domain consists of membrane spanning α helices and a channel gate, and Intracellular domain (I) has a single large α-helix for each subunit, each region separated by horizontal bars. MIR = Main Immunogenic Region, a special region located at the extracellular apex of each α subunit and constituting a major binding site for antibodies critical in autoimmune diseases like Myasthenia Gravis. Adapted from [52] with permission from Elsevier.

Each subunit of nAChRs comprises three major regions: an Extracellular domain (ECD) with N-terminal α-helix and multiple β-sheets forming the loop regions, a Transmembrane domain (TMD) with four membrane-spanning α-helices (M1–M4) housing the channel gate, and an Intracellular domain (ICD) with a single large α-helix that mediates cellular communications (Figure 1) [41,47]. The binding sites for agonists and competitive antagonists are located on the extracellular ligand-binding domains at the interface between adjacent subunits. The muscle-type nAChRs, e.g., (α1)_2_β1δγ, have two acetylcholine binding sites at the interface between α-γ and α-δ subunits [48]. The same applies to heteromeric neuronal nicotinic subtypes, e.g., α4β2, α3β4, and α9α10 nAChRs, where the binding pockets are formed by the contribution of the principal and complementary subunits. For neuronal homomeric receptors like α7 and α9 nAChRs, five acetylcholine binding sites have been identified at the interface between two α subunits [49]. The binding of acetylcholine and other agonists causes conformational changes in their structure, resulting in channel opening. The positively charged quaternary ammonium group of acetylcholine engages in cation-π interaction with multiple aromatic residues forming the aromatic cage, with tryptophan (W149) residues making a strong primary contribution, supported by tyrosine (Y93, Y190, Y198) residues that collectively shape and stabilize the binding pocket. The vicinal disulfide-linked cysteines (C192/193) from the principal α subunit contribute to structural architecture of the binding site region, while acidic residues such as aspartate and glutamate (positions corresponding to D174/180 and E183/189 in some receptor subtypes) from complementary non-α subunits contribute to the receptor function through electrostatic interactions and intersubunit hydrogen bonding that influence both ligand binding and channel gating [50,51,52,53]. These findings were derived primarily from site-directed mutagenesis studies of adjacent cysteines and other critical amino acid residues [54]. Recent advances, including the determination of the X-ray structure of Acetylcholine binding protein (AChBP) at atomic resolution, offer critical insights into ligand binding requisites of nAChRs [30].

## 3. Structural Homologs of nAChRs

The central approach to understanding these receptors involves determining the high-resolution atomic structure of a complete receptor or its truncated ligand-binding domain [40]. In the absence of a complete structure, a closely related structural homolog that can serve as a scaffold or template for chimeric constructs offers a practical alternative for studying nAChRs with greater depth. Such homologs can be categorized hierarchically based on their structural similarity to nAChRs and practical utility for chimera design, ranging from distantly related bacterial channels to highly homologous soluble binding proteins [55,56,57,58].

### 3.1. Bacterial Homologs

At the most distant evolutionary relationship, Erwinia ligand-gated ion channels (ELIC) function as a prokaryotic homolog of vertebrate pentameric ligand-gated ion channels (pLGICs) [59]. Although ELIC receptors lack an N-terminal α-helix, cysteine loop, or intracellular domain, they share key structural features with Cys-loop receptors, including the pentameric assembly and transmembrane organization. ELIC exhibits over 60% sequence homology within the transmembrane M2 region, the pore-lining segment essential for ion permeation. This similarity makes ELIC a valuable structural template for exploring the channel-gating mechanism of nAChRs, particularly investigating how structural changes in the TMD influence receptor function [60,61]. However, as integral membrane proteins like native nAChRs, the ELIC construct presents significant difficulties in cell surface expression, necessitating complex and costly membrane protein methods, which are more suitable for pharmacological characterization [62].

### 3.2. Cys-Loop Receptor Homologs

Among receptors with closer structural similarity to nAChRs, members of the Cys-loop receptor family, such as Gamma-aminobutyric acid (GABA_A_), Glycine, Serotonin (5-HT_3_), and nicotinic receptors, share structural similarities, including the common pentameric structures and domain organization [36]. Serotonin receptors (5-HT_3_), a cation-selective LGICs, share structural similarity with nAChRs at the sequence level, offering themselves as attractive structural and functional homologs enabling chimera design strategies. Both receptors share an extracellular αβ sandwich fold with a conserved series of β sheets (β1–10), a signature Cys-loop, and similar aromatic residues (Trp183 and Tyr234) for ligand recognition in orthosteric ligand binding sites. Four α-helical segments (M1–M4) in transmembrane domains share high sequence similarity in the pore-lining region. The less conserved intracellular domain in both members consists of the M3–M4 loop, which allows functional swap in chimera design [63,64,65]. The domain swap between nAChRs and 5-HT_3_ receptors can explain how specific domains contribute independently to receptor function, protein folding, and interaction with chaperones like Resistant to inhibitors of cholinesterase-3 (RIC-3), Transmembrane inner ear (TMIE) protein, which helps to localize allosteric binding sites that modulate channel gating and give insights into ligand selectivity [66]. Their membrane-associated nature [67] also favors their applicability to pharmacological profiling of nAChRs.

### 3.3. AChBP, a Soluble Homolog

**Figure 2:**
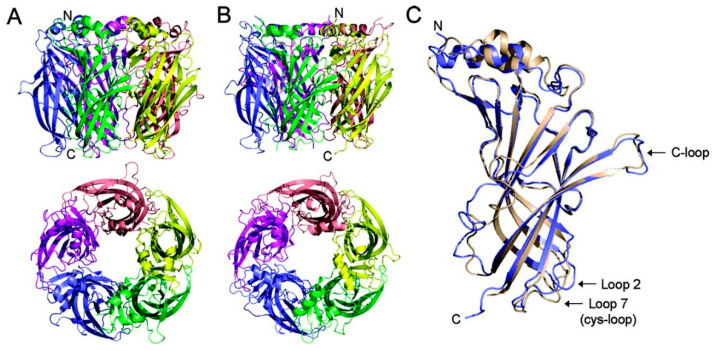
Crystal Structures of Bt-ACHBP and Ls-AChBP. Side and Top views of pentameric Bt-AChBP (**A**) and Ls-AChBP (**B**), extracellular domains of each monomer distinguished by separate colors. N indicates the amino terminal, and C indicates the Carboxy terminal. (**C**) Side view showing superimposed single monomers of Bt-AChBP (wheat) and Ls-AChBP (blue) sharing a similar structural scaffold [77].

At the highest level of structural applicability, a naturally occurring Acetylcholine binding protein serves as a closely related soluble homolog of the ligand-binding domain of nAChRs [68]. Its functional and structural similarity to nAChRs was confirmed by electron microscopy structure of Torpedo muscle type acetylcholine receptors at 4 Å resolution [11]. AChBP obtained from distinct species, such as freshwater snail *Lymnaea stagnalis* (Ls-AChBP), *Bulinus truncatus* (Bt-AChBP), and *Aplysia californica* (Ac-AChBP), is a naturally occurring soluble homolog for the ligand-binding domain of nAChRs. Comparative analysis of these three binding proteins showed highly similar binding pockets, particularly within aromatic cavities, and shared common features in their protein ligand interactions [69,70,71]. The homopentameric structure of AChBP (Figure 2) consists of an N-terminal α-helix followed by a β-sandwich and a disulfide bridge (Cys123–Cys136). Like nAChRs, AChBP consists of Loops A to F, forming a single binding pocket at subunit interfaces where Loops A, B, and C function as a principal side and Loops D, E, and F function as a complementary side. However, the Cys-loop segment in AChBP exhibits structural distinctions compared to the same region in native membrane receptors. Specifically, the segment connecting the two conserved cysteines (residues 123–136 in AChBP, corresponding to 128–142 in the Torpedo nicotinic receptor) is typically one amino acid shorter. Furthermore, where this segment is a conserved hydrophobic region in the native nicotinic receptors, interacting with the lipid membrane, it is mostly hydrophilic in AChBP. In AChBP, the loop is positioned on the membrane-facing side, and it is proposed that this region in the native receptor interacts directly with the transmembrane domain, a region that is structurally absent in the soluble AChBP structure [24,72,73,74]. Despite lacking TMD and ICD, AChBP mimics the pentameric assembly of nicotinic subunits, sharing high structural similarity with the ligand-binding domain in the extracellular region, even though they only exhibit 20–24% sequence identity [24,75]. It displays similar pharmacological profiles to those of nAChRs, mainly α7 subtypes, since the ligand-binding residues are conserved between AChBP and nAChRs [76]. For this reason, AChBP has been considered a valuable surrogate for nAChRs. Researchers engineer nAChR-AChBP chimeras to recapitulate native ligand binding properties while achieving the robust expression and stability required for high-resolution crystallographic studies [23].

## 4. Design and Engineering of Chimeras

The identification of structural homologs as a reliable surrogate system for investigating nAChRs (Section 3) prompts a shift towards practical considerations in chimera design. The engineering of chimeric receptors requires careful evaluation of structural compatibility alongside the preservation of functional attributes that closely resemble those of native receptors. Chimeras can be designed by following the domain substitution principle, which includes pairing the ECD, TMD, or ICD of one receptor with the corresponding domain of another receptor, by inserting specific loops known to influence ligand binding or gating mechanisms, and by combining human subunits with those from other species, like Torpedo, mouse, or snails [78,79]. Before modifications, it is essential to identify the region of conservation and variation. Conserved residues such as those crucial for ligand recognition and signal transduction, and structural integrity (A-F loops, β sheet scaffolds, glycosylation sites) are preserved from native receptors and have been identified from mutagenesis and affinity labeling studies for nAChRs. Regions where swapping is deemed essential for achieving the desired chimera’s properties, like hydrophilicity, require strategic swapping [20,23].

The extent to which structural modifications of native receptors to generate chimeras affect their intrinsic properties varies considerably, ranging from minimal to substantial effects, based on the swapped domains and their functional roles. For example, the α7-5HT_3_ chimera displays a pharmacological profile broadly similar to the wild-type α7, preserving the native rank order of potency while exhibiting a predictable shift in potency, altered efficacy, and slower desensitization kinetics arising from exchanged domains [11]. A similar minimal effect of structural engineering is seen when ECD of α7 was fused with Ls-AChBP; the resulting chimera retained native-like ligand recognition properties. This preservation in function occurred because key ligand-binding residues from the aromatic cage remained intact and properly oriented [80]. Conversely, modifications at the ECD-TMD interface can dramatically alter receptor properties. The ELIC-α7 chimera studies, in which α7 TMD was inserted to ELIC extracellular scaffold, revealed that even minor changes in the interface region were sufficient to abolish or strongly reduce sensitivity to several native α7 positive allosteric modulators acting through TMD, while the agonist binding remained relatively unaltered [81]. Taken together, these examples reveal a general pattern that preserving the local architecture of orthosteric binding site residues tends to maintain native-like pharmacology when structural changes were confined to surface-exposed binding loops, whereas swapping or modifying structural elements at domain interfaces and within the transmembrane region more strongly perturbs allosteric modulation, coupling, and gating behavior.

For the chimera design, the sequences between the target receptor, whose structure–function relationships are to be evaluated, and the well-characterized homologs are aligned using computational and sequence alignment tools to predict the structural compatibility between the sequences and 3D structural levels to minimize the risk of misfolding or loss of function [20]. The DNA sequence encoding the chimera receptor is then engineered by incorporating epitope (flag or histidine) tags at either the amino or carboxyl terminal for identification, detection, and purification, and signal peptides for membrane targeting, which is cloned into a suitable mammalian expression vector for robust expression. The sequence is verified by DNA sequencing to ensure correct assembly of the chimeric region. The functionality of the designed chimera is validated through subsequent cloning and surface expression in suitable host cells using immunostaining or related assays [82,83,84].

Functional validation of engineered chimera receptors is essential to confirm correct assembly, appropriate cellular localization, and ligand binding properties. For chimeras that lack a transmembrane ion channel domain, validation focuses on characterizing ligand binding rather than electrophysiological assays such as patch clamp or two-electrode voltage clamp [85,86]. Expression of chimera receptors is typically achieved in mammalian cell lines such as adherent cells (human embryonic kidney, HEK293; African green monkey kidney fibroblast Cells, COS-7; Chinese hamster ovary, CHO cells), and suspension cells (suspension-adapted HEK293 cells) or insect cells (*Sf9*). Native nAChRs are challenging to express in mammalian systems due to strict pentameric assembly, subunit-dependent stoichiometry, inefficient folding and trafficking, and dependence on specific chaperones to produce functional surface receptors. The chimeric receptor strategy effectively overcomes these limitations while preserving native receptor pharmacology. Following the expression, these receptors can be purified using chromatographic techniques for biochemical and biophysical characterization [87,88,89,90,91]. Receptor surface expressions can be confirmed and visualized using immunofluorescence microscopy or quantified with flow cytometry [92,93]. Standard analytical approaches, such as SDS-PAGE and Native-PAGE, immunoblotting, or mass spectrometry, are used to confirm that the receptor is produced and assembled correctly at the molecular level [94]. Comparing the binding characteristics of chimeric receptors to their wild-type counterparts is another critical validation step. This comparison confirms that structural engineering does not significantly alter the function of the ligand-binding domain. Radioligand binding assays help determine key parameters like total number of binding sites (maximum binding capacity, *Bmax*) and affinity of the ligand for the receptor (dissociation constant, *Kd*) [95]. Alternatively, label-free analysis of ligand interactions with chimera receptors can also be performed utilizing Surface Plasmon Resonance (SPR) or Isothermal Titration Calorimetry (ITC), providing real-time analysis data for association and dissociation rates as well as thermodynamic parameters of binding [96,97,98]. These binding assays collectively demonstrate that the functional elements of the chimera are operating correctly as designed. When combined with structural approaches such as X-ray crystallography or cryo-EM techniques, these methods provide a comprehensive characterization of binding properties and conformational states of chimera constructs [25,99].

## 5. Application of the Chimera Strategy

Following the design principles and validation methods detailed in the previous section, we now examine how chimeric receptors have been applied in addressing key biological questions concerning nAChRs. The application of chimeric receptors grew as scientists sought to determine how different subunits contributed to receptor assembly, stoichiometry, function, and pharmacology. Heteropentameric nAChR is composed of distinct subunits. The assembly of these subunits into a functional receptor is guided by certain regulatory regions within each subunit. For example, subunit association in Cys-loop receptors is regulated by the conserved pair of cysteine residues linked with disulfide bonds in the N-terminal extracellular domain of each subunit [100,101]. The subunit assembly and interactions for muscle-type nAChRs were studied by constructing a chimera between the N-terminal domain of the δ subunit fused to the rest of the γ subunit and vice versa. This study suggested that initial subunit association and interaction are mediated by respective N-terminal and C-terminal regions of the γ subunit [102]. In addition to comprehending fundamental assembly principles, chimeric methodologies have proven essential for dissecting more complex functional relationships. For instance, the correlation between the number of functional binding sites and maximal receptor activation has been determined using a chimeric receptor approach. Andersen N et al. (2013) [103] engineered high-conductance and low-conductance α7 subunits to study the receptor occupancy and channel open time relationship. This study revealed that, unlike wild-type α7, a chimeric receptor in which the transmembrane domain of α7 was replaced with that of the 5-HT3A receptor required multiple occupied binding sites for maximal activation. This finding demonstrates that altering the transmembrane domain changes the stoichiometry necessary for full activation, indicating complex interdependence between receptor domains [103]. Chimeras also allow the introduction of specific mutations to enable detailed kinetic analysis. Rayes D et al. (2005) introduced mutations in the M3–M4 cytoplasmic linker of α7-5HT_3A_ chimera without affecting its affinity and potency for agonists to study the role of M3–M4 linker in the channel-gating mechanism [104].

Chimeric constructs have been fruitful for dissecting the complex multi-subunit receptors, like nAChRs, where traditional mutagenesis attempts are limited. These have proven to be significantly beneficial in investigating ligand-receptor interaction at both orthosteric and allosteric binding sites. The α7 chimera constructed by combining the ECD of human α7 and *Ls*-AChBP facilitated the identification of the high-resolution crystal structure of α7 nAChRs ligand-binding domain, further disclosing molecular rearrangements and interactions involved in agonist recognition [20]. Identification of binding sites for agonists, competitive antagonists, and modulators explains the molecular mechanism of receptor activation and can provide evolutionary insights for residues conserved across species [105].

**Figure 3:**
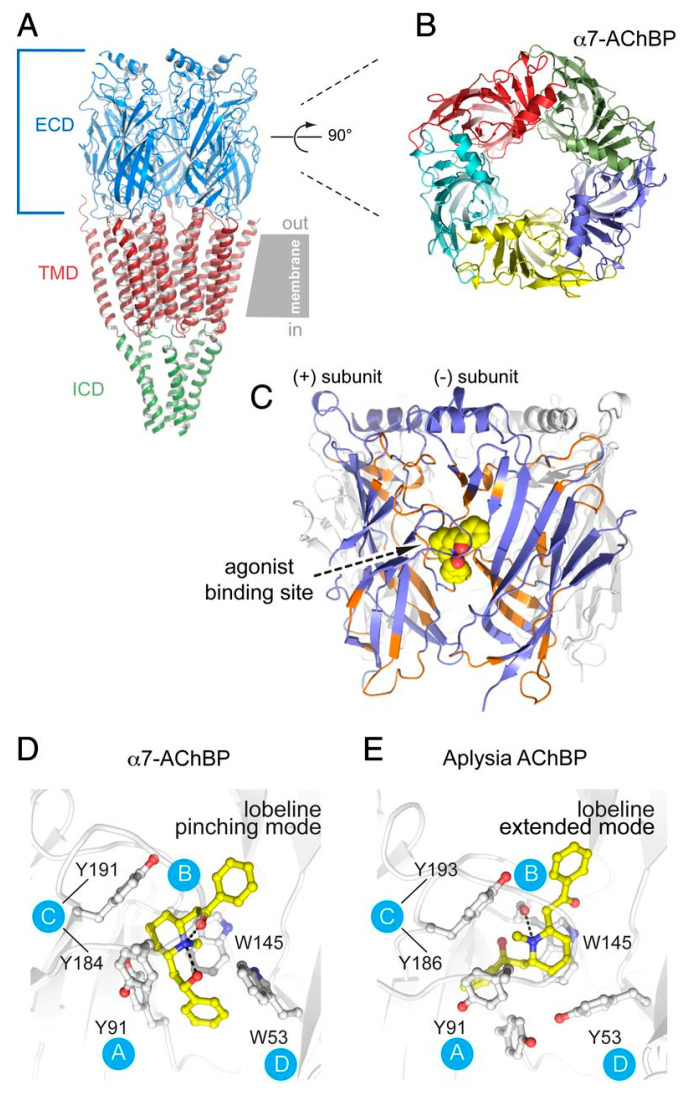
Structural details of α7-AChBP chimera and agonist binding sites. (**A**) Side view of torpedo nAChR representing Extracellular (ECD), Transmembrane (TMD), and Intracellular domain (ICD) with respective blue, red, and green colors. The dashed line and arrow indicate a 90° rotation around the pore axis. (**B**) Top-down view of α7-AChBP complexed with agonist lobeline after 90° rotation. (**C**) Side view of agonist binding site at the interface between principal (+) and complementary (−) subunits of α7-AChBP complexed with lobeline (yellow sphere). Blue regions in the chimera correspond to native α7 residues, and orange regions denote AChBP residues. (**D**,**E**) Agonist binding sites highlighted with different poses of lobeline in α7-AChBP chimera (pinching mode) and Aplysia AChBP (extended mode), respectively. Blue circles indicate binding site loops, ball and stick representations are for conserved residues in the binding site, and the dashed lines indicate hydrogen bond [21].

The α7-AChBP design has been utilized to identify molecular determinants not only at orthosteric but also at allosteric binding sites of α7 nAChRs. Figure 3 illustrates the binding of agonist lobeline to the orthosteric sites of α7-AChBP, which stabilizes the tweezer-like conformational states through its interaction with conserved aromatic residues, and aids in elucidating how ligands are recognized by the receptor. Three allosteric binding sites, including the N-terminal alpha helix, the vestibule pocket opposite the orthosteric sites, and the sub-agonist pocket right below the orthosteric sites, have been identified for α7 nAChRs. This information has expanded opportunities for discovering novel allosteric modulators for the serious implications of α7 nAChRs [21]. Positive Allosteric Modulators (PAMs) of α7 nAChRs also bind at the TMD. Based on the suspected role of the ECD-TMD interface in receptor interaction with allosteric modulators, multiple ELIC-α7 nAChR chimeras were designed by fusing the TMD of human α7 nAChRs with the ECD of ELIC. This study suggested that an ELIC-α7 nAChR chimera resembling an α7 nAChR ECD-TMD interface showed potentiation by PAMs similar to native α7 nAChRs. The mechanism of allosteric potentiation was mediated by the ECD-TMD interface and thus imposes a strict structural requirement compared to the agonist-mediated activation [81].

The use of chimeric constructs extends beyond structural studies to address practical challenges in receptor pharmacology. A particularly illustrative example is the α6 nAChR chimera, which overcomes the challenges in expressing and characterizing α6-containing nAChRs, critical for nicotine reinforcement but difficult to express in heterologous systems like *Xenopus* oocytes. The α4/6 chimera, consisting of transmembrane and intracellular domains of α6 and the extracellular domain of α4 subtypes, bypasses α6’s poor expression in vitro, allowing pharmacological profiling of α6-containing receptors. This chimera, while retaining the α6-specific channel properties, provides a template for screening α6-selective antagonists with potential therapeutic applications in nicotine addiction [106]. The chimera receptor approach has gained popularity in determining receptor selectivity for both agonists and antagonists. High-affinity interactions between nAChRs and natural toxins, e.g., α-Bungarotoxin, have been studied using an α7-AChBP chimera complex. This structure has resolved the conserved residues governing toxin specificity in receptor subtypes, guiding the development of a selective antagonist for pain management and neurological disorders [107]. Furthermore, a chimera constructed by combining the N-terminal regions from β4 with the C-terminal regions from β2 revealed that specific regions of the β subunit are responsible for differences in agonist selectivity between β2- and β4-containing nAChRs, indicating that β subunit regions, not just the α subunit, contribute to the pharmacological properties of nAChRs [108].

Nonetheless, the application of chimera constructs in high-throughput screening to understand the role of nicotinic receptors in pathophysiology is evident. Human α7 nAChRs engineered with the mouse serotonin 5-HT_3_ receptor were expressed in mammalian cell lines. This chimera combines the N-terminal domain of the human α7 nAChRs with the transmembrane and C-terminal domains of the mouse serotonin 5-HT_3_ receptor and displays similar pharmacological properties to those of the native α7 subunits, allowing rapid screening of compounds for receptor selectivity and activation through an allosteric mechanism [11,66]. Chimeras have been generated by adding the ICD of three-domain eukaryotic Cys-loop (5-HT_3A_) receptors to two-domain prokaryotic (GLIC) receptors, to study the structure and function of ICD. The Cys-loop receptor ICD functions as a structural and functional modular unit, which can be deleted or replaced with heterologous sequences without disrupting the receptor’s ability to fold and assemble into a functional ion channel and respond to ligand binding. The ICD modularity enables the engineering of chimeras, such as GLIC-5-HT_3A_ hybrids, where prokaryotic ICDs are swapped with eukaryotic counterparts to study trafficking, gating mechanisms, and interactions with other proteins. This study suggested that the interaction of chaperone, RIC-3, requires the presence of ICD in the Cys-loop receptors [78].

Chimera constructs were used to identify allosteric binding sites in other Cys-loop receptors as well. A prokaryotic-eukaryotic chimera was designed by fusing the ECD from GLIC, a prokaryotic homolog from *Gloeobacter violaceus,* with the TMD from the GABA_A_ receptors α1-subunit to study the structure of γ-aminobutyric acid receptors (GABA_A_Rs). The α subunit is the critical subunit of GABA_A_ receptors, where its TMD houses the allosteric binding sites for neurosteroids. This GABA_A_-GLIC chimera enabled the precise identification of a structural location for the binding site of both potentiating and inhibitory neurosteroids, which were found to be at the lipid interface and the ion channel pore, respectively. This construction provided information on receptor function and modulation, facilitating the development of therapeutic agents selective for these sites [109].

## 6. Summary

A summary of the applications discussed in Section 5, along with key details on chimera construct design for nAChRs and associated challenges or success factors, structural characterization, and experimental validation, is presented in Table 1.

## 7. Conclusions and Limitations

This review has detailed how the chimeric receptor approach has revolutionized nAChRs research via its extensive applications. Here, we summarize its applications in a broader context and consider how the chimera strategy has impacted other Cys-loop receptor family members and future drug discovery efforts. Chimeric constructs are designed to overcome the challenges in protein expression, misfolding, stability, and solubility associated with their native form. They can reveal characteristic binding interfaces and conformational changes triggered by the binding of an endogenous or exogenous ligand [70]. This strategy has demonstrated success not only with nAChRs but also with other Cys-loop receptor family members. For instance, the chimera strategy was used to determine the accurate subunit stoichiometry of GABA_A_ receptors. Tretter V et al. (1997) [113] identified the ratios of respective subunits of multimeric GABA_A_ receptors by a thorough study of the relative reactivity of subunit-specific antibodies to chimeric proteins composed of the N-terminal domain from one subunit and remaining regions from other subunits. This study resolved controversies of GABA_A_ receptors composition by providing evidence for pentameric assembly of four alternate α and β subunits connected by one γ subunit in the ratio 2α:2β:1γ [113].

Turning to drug discovery challenges, this process is often impeded by insufficient structural information about receptors, their binding sites, and binding mechanisms, especially for late-discovered receptors that are highly unstable, exhibit low or no expression, or show a lack of crystal interactions [114]. One such example is the α9α10 nAChR subtypes distinctly localized in the cochlear outer hair cells, where they mediate efferent synaptic transmission from medial olivocochlear fibers [115]. Studies have suggested their role in normal auditory function, while their degeneration is directly linked to hearing disorders [116,117,118]. A chimeric α9α10 nAChR, engineered by fusing the ligand-binding domain of α9 or α10 subunits to the C-terminal domain of 5-HT_3A_, has been developed to study the radioligand binding properties [111], reflecting the possibility of creating more of such chimeras to further investigate their structure–function relationship and identification of allosteric modulators that can compensate for the loss of receptors to help prevent hearing implications [119,120]. While the examples presented throughout this review highlight numerous successes, it is essential to acknowledge that the chimeric approach, like any other experimental strategy, has certain limitations that must be considered when interpreting structural and functional data.

To summarize the strategic value of this approach, chimeras offer advantages to enable functional expression of otherwise intractable receptor subtypes, analyze specific domain contributions to receptor pharmacology and channel gating, and facilitate screening and development of subtype-selective ligands and antagonists. Despite these advantages, the chimeric approach has inherent limitations that necessitate careful consideration. AChBP-based chimeras lack transmembrane and intracellular domains, potentially missing critical interdomain allosteric communications, lipid interactions, and associations with scaffolding proteins, which are essential for understanding receptor regulations in the native cellular contexts. As a result, chimeric constructs between AChBP and receptor domains may not fully reproduce the conformations of the native receptor or its regulatory mechanisms, potentially limiting their ability to model physiological receptor behavior accurately [121]. Introducing chimeric modifications can disrupt receptor expression or compromise receptor function, requiring the construction of multiple chimeric variants, considering the specific requirements of different expression systems [122]. Other limitations of chimeric constructs may include protein misfolding, altered receptor trafficking and localization, or even altered ligand interactions [123]. These limitations, however, should not be viewed as insurmountable obstacles but as considerations that inform experimental design. When appropriately designed with awareness of these constraints, chimeric constructs remain powerful tools that have advanced our understanding of nAChRs and related receptors.

## 8. Future Directions

Integration of chimeric receptor strategy with advanced structural methods, including X-ray diffraction and cryo-EM, provides a detailed structure of receptor complexes, enabling visualization of the precise conformational changes and domain arrangements that occur as the receptor transitions between different functional states, ultimately revealing the molecular mechanisms underlying receptor function and regulation. Expanding the chimera studies could illuminate orthosteric and allosteric binding sites, receptor modulation, assembly, and trafficking mechanisms, and synaptic localization mediated by scaffolding proteins. Rational design of chimeras can accelerate high-throughput screening to study structure–activity relationships and optimize lead candidates to discover novel subtype-selective drugs targeting nAChRs involved in neuropsychiatric disorders and addiction. The chimera approach and its application in high-throughput screening remain a significant area of research to bridge gaps between structural biology, pharmacology, and therapeutic development. Looking ahead, the integration of chimeric and computational approaches holds the greatest potential for advancement. Current advances in computational techniques and artificial intelligence (AI), such as the development of AlphaFold for protein modeling, are rapidly transforming receptor biology through more accurate structure predictions, ligand screening, and drug discovery. Recent studies, e.g., Ng et al. (2018) [124], have demonstrated the powerful synergy between nicotinic receptor chimera and machine learning approaches by predicting ligand binding and addiction potential for thousands of tobacco constituents. This highlights a promising future direction for incorporating structural biology and AI in receptor research.

Far from rendering chimeric approaches obsolete, AlphaFold complements them by addressing distinct challenges in nAChR structural studies. AlphaFold has demonstrated exceptional proficiency in predicting monomeric protein structures with high accuracy for single-domain proteins [125] and has been successfully applied to membrane proteins, including ion channels and receptors [126,127]. More recently, AlphaFold3 has expanded these capabilities to predict protein–protein and protein–ligand interactions through a diffusion-based approach, offering new opportunities to model receptor complexes, to identify candidate ligand-binding regions, domain interfaces, and allosteric sites, and to guide rational design of chimeric receptors by highlighting structurally compatible regions [128,129]. However, several fundamental challenges remain specific to nAChR biology that necessitate continued use of experimental chimeric approaches. While AlphaFold multimer shows promise, the prediction of correct pentameric assembly and subunit arrangement for heteromeric nAChRs with multiple stoichiometries, e.g., (α9)_2_(α10)_3_ versus (α9)_3_(α10)_2,_ remains challenging [130,131]. Experimental chimeras provide crucial evidence about which subunits form functional interfaces and which arrangements are physiologically relevant. Proteins are dynamic and can exist in multiple conformation states, resting, active, or desensitized states, with function tied to interconversion between them. AlphaFold predicts a single static structure, not a full ensemble of the conformational diversity of proteins [132,133], while chimeric approaches using ligand-bound crystallography capture discrete functional conformations. Current prediction algorithms struggle to predict the effect of point mutations and posttranslational modifications, especially in membrane proteins, and do not explicitly model lipid–protein interactions, which is critical for nAChR TMD function [133,134]. Important challenges also remain for disordered regions and multi-state systems, where function depends on subtle allosteric changes [128].

AlphaFold is significantly dependent on an extensive PDB dataset of experimentally validated structures to train its predictive models [135]. Predicted structures, while useful in generating hypotheses in drug discovery and mechanistic studies, do not replace experimental approaches [136]. Chimeric receptor systems remain essential for experimental validation for direct assessment of ligand binding, receptor function, conformational change, and the connection between structural prediction and actual receptor activity and gating mechanisms [36,137]. It is therefore most accurate to view AlphaFold as complementary to experimental approaches, especially for modeling large protein complexes to reveal receptor dynamics and interfaces that refine, constrain, or correct AlphaFold models [138,139]. The integration of computational modeling with chimera-based structural prediction and functional studies provides a robust strategy to advance understanding of nicotinic receptor biology and to support rational drug discovery. Both approaches working in concert, rather than one replacing the other, represent the optimal path forward for the field.

## Figures and Tables

**Table 1:** Summary of Chimera composition and its application in receptor biology.

S.N.	Chimera Composition	Structural Method and Resolution	Key Technical Details and Challenges	Application
i.	α7 nAChR/*Ls*-AChBP chimera, free (PDB code: 3SQ9) and Epibatidine bound (PDB code: 3SQ6)	X-ray Crystallography (Apo—3.10 Å Epi bound—2.80 Å)	Construct design: N-terminal and Cys-loop of AChBP fused with the remaining ECD of α7; 64% sequence identity with native α7 nAChRs. Expression: *Pichia pastoris* Challenges: Native α7 ECD failed to express in yeast; multiple chimera iterations were required. Success factors: Strategic inclusion of AChBP segments improved expression; Epi stabilized crystals diffracted better. Validation method: Radioligand binding	First high-resolution structure of α7 ligand binding domain: Mechanistic insight into agonist binding, signal transduction, template for drug design targeting α7 nAChRs [80]
ii.	α7 nAChR/AChBP chimera complex with α-BTX (PDB code: 4HQP)	X-ray Crystallography (3.51 Å)	Construct design: same as entry (i) Challenge: Glycan chains interfered with crystal formation, requiring Endo HF deglycosylation to obtain diffracting crystals Success factor: Chimera expressed stable pentamers and bound α-BTX with high affinity. Comparison with the agonist-bound structure 3SQ6 showed that α-BTX binding locks loop C in a uniformly open conformation, in contrast to the closed-in loop C conformation observed with epibatidine. Validation method: Radioligand binding	Understanding toxin interaction with α7 subtypes and comparison with agonist-bound structure [107]
iii.	α7 nAChR/Ls-AChBP chimera (PDB code: 5OUH, 5OUG, 5OUI)	X-ray Crystallography (2.5–3.10Å)	Construct design: same as entry (i) Expression: *Sf9* insect cells Challenges: PAMs’ binding site initially cryptic, required screening of a large fragment collection Success factors: Automated collection of diffraction data from thousands of fragments-soaked crystals Validation method: Surface Plasmon Resonance (SPR) Electrophysiology	Identification of a novel allosteric binding site for PAMs [25]
iv.	α7 nAChR/AChBP chimera (PDB code: 5AFH, 5AFJ, 5AFK, 5AFL, 5AFM, 5AFN)	X-ray Crystallography (2.15–2.85Å)	Construct design: same as entry (i) Fragment library screening: Tested diverse chemical scaffolds. Expression: *Sf21* insect cells Challenges: Low fragment affinity Success factors: Lobeline co-crystallization helped stabilize the structure and revealed an induced fit mechanism. Validation method: SPR Electrophysiology	Identification of three allosteric binding sites in the ECD of α7 nAChRs and associated conformational dynamics utilizing a fragment-based drug screening approach [21]
v.	α7 nAChR/5-HT_3_ receptor chimera	ND	Construct design: N-terminal part of α7 combined with the C-terminal region of 5-HT_3_ subunit. Challenges: Several chimeras had to be built with optimization of junction residues Success factor: At least one construct expressed a functional receptor with α7-like nicotinic ligand binding properties and 5-HT_3_-like channel properties, potentiated by external Ca^2+^ ions. Validation method: Electrophysiology, Radioligand binding	Demonstrated the possibility of fusing ECD of α7 nAChRs with TMD and ICD of serotonin receptors for functional expression and identifying molecular determinants for ligand binding and channel gating in the α7 nAChR subtype [110]
vi.	Human α7 nAChR/mouse 5-HT_3_ receptor chimera	ND	Chimera construct: ECD of α7 fused with TMD and the C-terminal region of 5-HT_3._ Expression: HEK-293 cells Challenge: Wild-type α7 exhibited poor, stable expression Success factors: Stable cell line development, 129 chimera clones screened for functional expression, clones with large Ca^2+^ response selected for imaging. Validation method: Electrophysiological assay Fluorescent binding assay High-throughput Calcium Imaging	Stable mammalian expression, a tool for functional high-throughput screening [11]
vii.	Rat α9 or α10 nAChR subunit/mouse 5-HT_3A_ receptor chimera	ND	Chimera construct: ECD of α9 or α10 fused to the C-terminal region of 5-HT_3A_. Expression: HEK-293 cells, *Xenopus laevis* oocytes Challenge: Mammalian cells transfected with native α9 or α10 cDNAs alone or expressed showed no expression Success factor: Successful co-expression of α9 and α10 chimera Validation method: Electrophysiology Radioligand binding assay	First functional expression of α9 and α10 receptors alone in mammalian cell lines and their co-expression, to dissect the role of each subunit in ligand binding; model to study native α9α10 nAChRs [111]
viii.	ELIC/α7 nAChR	ND	Chimera Construct: Multiple chimeras generated between ELIC (ECD) and α7 (TMD), with systematic swaps of interface elements. Challenge: Most constructions showed no response Success factor: Only the construct preserving specific interface residues in α7 showed PAM potentiation. Validation method: Electrophysiology	Role of ECD-TMD interface in allosteric modulation [81]
ix.	α6/α4 nAChR chimera	ND	Chimera Construct: ECD of α6 litigated with the remaining parts of the α4 subunit. Challenge: α6 does not form a functional receptor with the β2 subunit Success factor: α6/α4 chimera injected together with β2, retained α6-specific properties while achieving functional expression. Validation method: Electrophysiology	Determination of amino acid residues driving selectivity of neurotoxin, α-conotoxin BuIA, between α6 and α4 subunits [106,112]
x.	β4/β2 nAChR chimera	ND	Chimera construct: N-terminal of β4 combined with C-terminal of β2 and co-expressed with α3 in oocytes. Challenge: Required systematic testing of multiple chimera designs Success factor: Identified regions of β subunits critical for agonist sensitivity, revealed that β subunits actively contribute to receptor pharmacology, not just the α subunit. Validation method: Electrophysiology	Investigating interface contributions to ligand binding, subtype pharmacology, and ligand selectivity [108]

Abbreviations: α-BTX, α-Bungarotoxin; Apo, Apoprotein (Unliganded); cDNA, complementary DNA; Endo HF, Endoglycosidase HF; Epi, Epibatidine bound; HEK-293, Human Embryonic Kidney 293 cells; ND, Not Determined; PDB, Protein Data Bank; Sf9/Sf21, *Spodoptera frugiperda* (insect cells, lines 9 and 21).

## Data Availability

No new data were created or analyzed in this study. Data sharing is not applicable to this article.

## Abbreviations

5-HT_3_	5-hydroxytryptamine type 3 receptor or Serotonin type 3 receptor
*Ac*-AChBP	*Aplysia californica* Acetylcholine Binding Protein
AChBP	Acetylcholine Binding Protein
*Bt*-AChBP	*Bulinus truncatus* Acetylcholine Binding Protein
Cryo-EM	Cryo-Electron Microscopy
ECD	Extracellular Domain
ELIC	Erwinia Ligand Gated Ion Channel
GABA	Gamma-Aminobutyric Acid
GLIC	Gloeobacter Ligand-gated Ion Channel
ICD	Intracellular Domain
LGICs	Ligand-gated Ion Channels
*Ls*-AChBP	*Lymnaea stagnalis* Acetylcholine Binding Protein
nAChRs	Nicotinic Acetylcholine Receptors
NMR	Nuclear Magnetic Resonance
PAMs	Positive Allosteric Modulators
pLGICs	Pentameric Ligand Gated Ion Channels
RIC-3	Resistant to inhibitors of cholinesterase-3
TMD	Transmembrane Domain
TMIE	Transmembrane Inner Ear
